# Primary malignant peripheral nerve sheath tumour of the trachea: a case report and literature review

**DOI:** 10.1186/s13019-020-01285-x

**Published:** 2020-09-11

**Authors:** Yan Hu, Siying Ren, Wei Han, Boyou Zhang, Lu Shu, Yi Sun, Fenglei Yu, Wenliang Liu

**Affiliations:** 1 Department of Thoracic Surgery, Second Xiangya Hospital of Central South University, No. 139 Renmin Road, Changsha, 410011 China; 2 Department of Respiratory and Critical Care Medicine, Second Xiangya Hospital of Central South University, Changsha, 410011 China; 3 Department of Pathology, Second Xiangya Hospital of Central South University, Changsha, 410011 China

**Keywords:** Malignant peripheral nerve sheath tumour, Trachea, Misdiagnosis

## Abstract

**Background:**

Malignant peripheral nerve sheath tumours (MPNSTs) of the trachea are extremely uncommon neoplasms with unknown genetic and clinical profiles. Only individual cases have been reported in the literature to date.

**Case presentation:**

Here, we present a rare case of a 61-year-old female patient with a primary MPNST of the trachea who complained of irritating cough and progressively increasing breathlessness for 4 weeks. This patient initially underwent intraluminal resection of the mass and was misdiagnosed with clear cell sarcoma. Less than a year later, the mass relapsed, and the obstructive symptoms reappeared and gradually worsened. Debulking of the endotracheal tumour mass was performed once again, and an MPNST was definitively diagnosed. Open sleeve tracheal resection and tracheoplasty were later performed with curative intent. This patient was alive without recurrence at her six-month postoperative follow-up. We also compared the clinical outcomes of previously reported cases of MPNSTs and our case.

**Conclusions:**

This paper emphasizes that thoracic surgeons should be aware that malignant peripheral nerve sheath tumours of the trachea can be misdiagnosed in clinical practice and must be included in the differential diagnosis of tracheal neoplasms.

## Background

Primary tracheal cancer is one of the rare malignancies in the upper airway, accounting for only 0.1–0.4% of all newly diagnosed tumours. Currently, there is no validated staging system for diagnosis, survival prediction, or management. The prognosis of patients diagnosed with tracheal cancer is dismal. MPNST commonly occurs in the extremities. Primary MPNST of the trachea has rarely been reported in the literature. We herein present a rare case of an MPNST in the trachea initially misdiagnosed as clear cell sarcoma and present a literature review of previously reported cases of tracheal MPNSTs.

## Case presentation

A 61-year-old female patient was admitted to a local hospital in August 2018 because of irritating cough and progressively increasing breathlessness over a duration of 4 weeks. Rinopharyngeal thoracic computed tomography scans showed severe luminal obstruction of the middle upper segment of the trachea caused by a mass. Rigid tracheoscopy was performed, revealing an endotracheal mass with a wide base originating in the posterior lateral wall of the trachea occluding 75 % of the lumen. The tumour was then endoscopically resected through high-frequency electric coagulation, cutting and argon plasma coagulation in that hospital. Postoperative pathology gave a misdiagnosis of clear cell sarcoma of the trachea. The patient was free of any discomfort until April 2019. Then, she complained of the reappearance of nonproductive cough and shortness of breath, which gradually worsened.

**Fig. 1:**
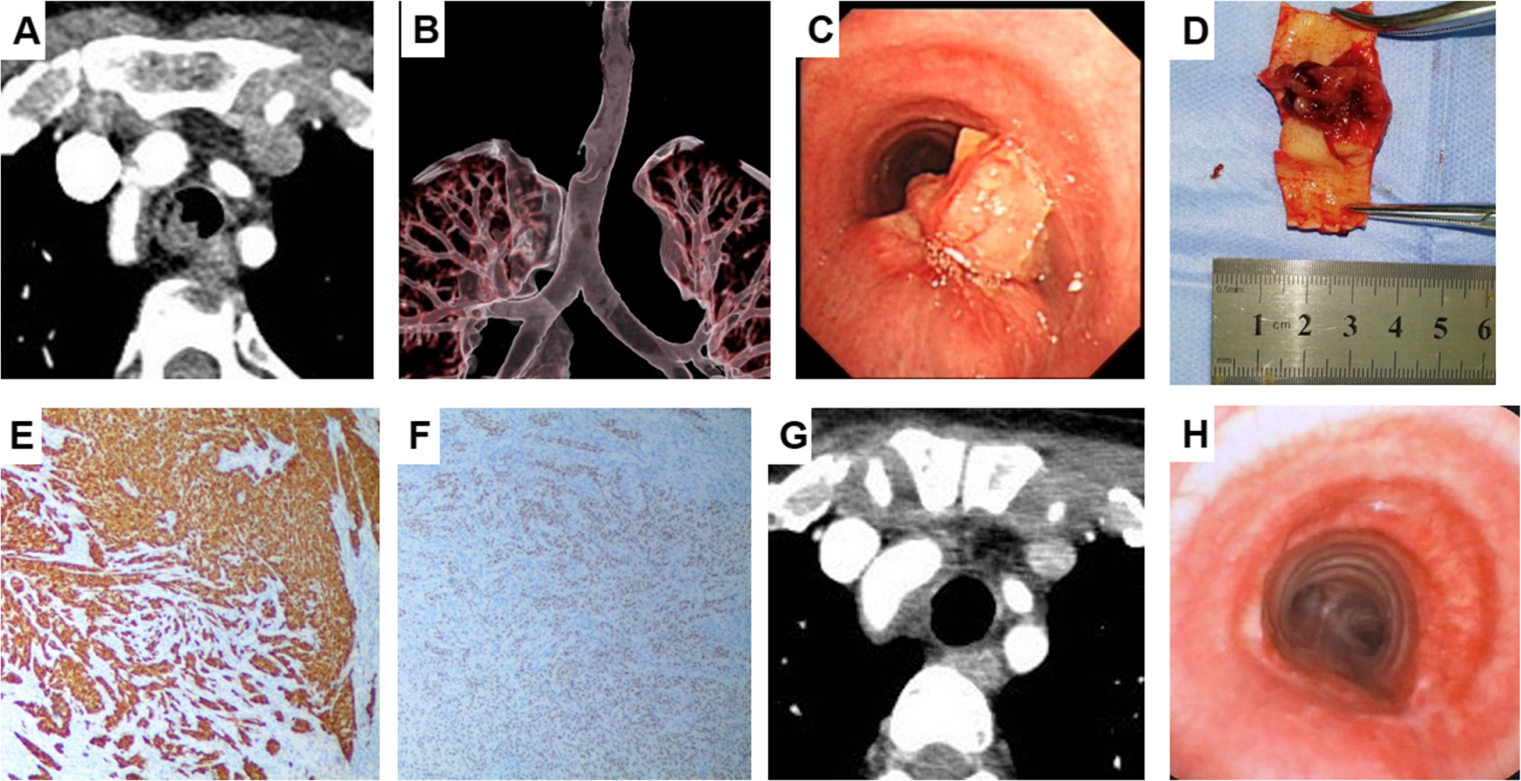
A case of a tracheal malignant peripheral nerve sheath tumour. **a** Preoperative thoracic CT scans reveal a tumour arising from the right posterior lateral wall of the trachea; **b** Reconstruction of the airway demonstrated a tracheal defect approximately 4.7 cm above the carina; **c** Bronchoscopy showed a cauliflower-like mass obstructing 60 % of the tracheal lumen; **d** View of the resected tumour; **e** The tumour cells are positive for S100; **f** The tumour cells are positive for SOX-10; **g**, **h** Postoperative thoracic CT scans and bronchoscopy at the six-month follow-up showed no local recurrence

This patient was subsequently admitted to our hospital in June 2019. Cervicothoracic CT scans revealed a 2.5 × 1.8 × 1.6 cm mass arising from the right posterior lateral wall of the trachea approximately 4.5 cm below the glottis and 4.7 cm above the carina with a suspicious extension of the oesophageal wall (Fig. 1). Bronchoscopy was performed, showing a cauliflower-like mass obstructing 60 % of the tracheal lumen. After obtaining biopsies, an in-site exfoliated cell smear was performed, which indicated suspicious tumour cells with nuclear atypia. Tumour debulking was then performed through high-frequency electric coagulation and cutting to alleviate airway obstruction. This patient gained obvious relief from the obstructive symptoms postoperatively.

Histopathology revealed diffuse spindle cells with pleomorphic nuclei and translucent cytoplasm. Mitoses and necrosis were noted. The tumour cells were positive for S100 (Fig. 1), SOX-10, and vimentin but negative for pancytokeratin, SMA, desmin, transcription factor E3 (TFE3), and master myogenic regulatory factor (MyoD1) by immunohistochemistry. Melanocytic-specific markers, such as HMB45 and melan-A, were also negative. Thus, taken together, the morphologic and immunohistochemical features favoured the diagnosis of a malignant peripheral nerve sheath tumour (MPNST). Owing to the epithelioid morphology and diffuse S100 immunoreactivity, an unusual variant of an epithelioid MPNST was therefore determined. The tumour of this patient was further classified as a sporadic MPNST since no related signs of neurofibromatosis type 1 (NF1) were noted.

Because of the rarity of this disease, this case was discussed in a multidisciplinary tumour board meeting. A consensus was reached, and this patient was later treated with tracheal resection and tracheoplasty through a median sternotomy approach. Intraoperative exploration did not find obvious neoplastic involvement of the oesophageal wall. The length of the resected trachea was approximately 3 cm. Both margins turned out to be negative. The airway was reconstructed with a posterior continuous suture and an anterior row of interrupted sutures, and the anastomosis was covered with autologous thymus tissue. This patient experienced an uneventful postoperative course. At her six-month postoperative follow-up, she continued to do well without complications or evidence of recurrence (Fig. 1). The patient is scheduled to receive regular follow-up evaluations every 6 months using computed tomography and bronchoscopy to monitor for recurrence.

## Discussion

Primary tracheal cancer is one of the rare malignancies in the upper airway, accounting for only 0.1–0.4% of all newly diagnosed tumours, with an incidence of 2.6 new cases per 1,000,000 people per year [1]. Owing to its rarity and the difficulties of diagnosis via chest X-ray, tracheal cancer is usually misdiagnosed. CT scans together with tracheobronchoscopy form the mainstays for diagnosing and staging primary tracheal cancer. Squamous cell carcinoma is the predominant histological type of tracheal cancer, followed by adenoid cystic carcinoma and neuroendocrine tumours [2]. Tracheal cancer, when large, typically presents with obstructive symptoms such as dyspnoea, stridor, wheezing, cough, and haemoptysis.

Currently, no validated staging system exists for diagnosis, survival prediction, or management. The prognosis of patients diagnosed with tracheal cancer is dismal, with 5-year and 10-year overall survival rates of 5–15% and 6–7%, respectively [3]. Patients with an old age, large tumour size, advanced tumour extension and no surgery may have a worse prognosis [4]. Primary tracheal cancer can be treated with surgery, endotracheal resection by various techniques, and radiotherapy [3]. Surgical resection is the treatment modality that offers the best chance of achieving a long-term cure. Endotracheal treatment is tailored to debulk the tumour mass for patients with acute airway obstruction prior to definitive surgery or as a palliative regimen in patients with unresectable disease. Radiotherapy is indicated as adjuvant therapy after surgical resection, as primary therapy in patients with inoperable or unresectable disease and as palliative therapy for severe symptoms.

Malignant peripheral nerve sheath tumours are highly aggressive neoplasms, accounting for 5 to 10% of soft-tissue sarcomas, and can be sporadic, be observed in patients with neurofibromatosis type 1, or develop post irradiation [5]. Owing to its morphological heterogeneity and lack of definitive markers, MPNSTs can be easily misdiagnosed as other malignancies that exhibit similar histologic features, such as a variety of sarcomas (synovial sarcoma, rhabdomyosarcoma, leiomyosarcoma, dedifferentiated liposarcoma, clear cell sarcoma, and dermatofibrosarcoma protuberans) as well as non-mesenchymal tumours (especially melanoma) [6]. Lehnhardt et al. showed that the false primary diagnosis rate of MPNST was 78.4%, which was the highest among false diagnosis rates of the eight most frequent sarcoma types [7]. A recent study showed that 18.1% (29 out of 160) of initial diagnoses of MPNST had to be revised during reassessment [8].

The pathogenic mechanism of MPNST remains to be determined. The loss of NF1 and p53 are considered the most frequently reported gene mutations in MPNST and are responsible for malignant transformations arising [9]. Accumulating evidence has indicated the existence of a multistep aggregation of additional mutations of multiple tumour suppressors, cell cycle dysfunctions, and signalling regulation genes, resulting in signalling cascade disorders and receptor tyrosine kinase amplification [10].

MPNSTs commonly occur in the proximal portions of the extremities. An MPNST arising primarily from the trachea, however, is rarely seen. To the best of our knowledge, only three cases of primary MPNSTs of the trachea have been described in the literature [11–13]. The clinicopathological features of the previously reported cases and our case are summarized in Table 1. Epithelioid MPNSTs, an uncommon histologic variant of MPNSTs, constitutes less than 5% of MPNSTs and features a distinctive epithelioid morphology and diffuse S100 immunoreactivity [14]. It is noteworthy that this patient was diagnosed with an epithelioid MPNST, which makes this case even more rare.

**Table 1:** Summary of clinicopathological characteristics

	Shah et al	Neumann et al	Nemade et al	Present case
Age/sex	63/male	30/ND	33/male	61/female
Symptoms	Dyspnoea	Dyspnoea	Dyspnoea, stridor	Cough, Dyspnoea
Smoking status	Yes	ND	No	No
Location	Thoracic trachea	Cervical trachea	Cervical trachea	Thoracic trachea
Tumour size (cm)	2.5	1.5	ND	2.5
Lymphovascular invasion	Yes	ND	ND	No
Treatment	ET, Surgery, RT	ET, Surgery	ET, Surgery	ET, Surgery
Resection margins	Negative	ND	Negative	Negative
Clinical outcomes	ND	free of disease for 6 years	free of disease for 32 months	free of disease for 6 months

*ET* Endoluminal treatment, *RT* Radiotherapy, *ND* Not described

## Conclusions

Herein, we reported a rare case of a tracheal malignant peripheral nerve sheath tumour with epithelioid morphology and reviewed the literature on MPNSTs of the trachea. This paper emphasizes that thoracic surgeons should be aware that malignant peripheral nerve sheath tumours of the trachea can be misdiagnosed in clinical practice and must be included in the differential diagnosis of tracheal neoplasms.

## Data Availability

As a case report, all data generated or analysed are included in this article.

## Abbreviations

MPNST: Malignant peripheral nerve sheath tumour
TFE3: Transcription factor E3
MyoD1: Master myogenic regulatory factor
NF1: Neurofibromatosis type 1
